# Supplementary material for: Non-Severe Hypophosphatemia in Older Patients: A Systematic Review
Source: Nutrients. 2025 Apr 16;17(8):1354. doi: 10.3390/nu17081354 (PMC12030319; doi:10.3390/nu17081354)
Supplement: Supplementary file 1 [file nutrients-17-01354-s001.zip › nutrients-3543033-supplementary.pdf]

Supplementary Materials

Search strategy

Pubmed:
(((hypophosphatemia[MeSH Terms]) OR (phosphorus supplementation)) AND ((elderly[MeSH Terms]) OR (older adult*))) NOT ((critical illness[MeSH Terms]) OR (care intensive unit OR ICU[MeSH Terms]))

Web of Science:
(((hypophosphatemia) OR (phosphorus supplementation)) AND ((elderly) OR (older adult*))) NOT ((critical illness) OR (care intensive unit OR ICU))

Scopus:
TITLE-ABS-KEY ((((hypophosphatemia) OR (phosphorus AND supplementation)) AND ((elderly) OR (older AND adult))) AND NOT ((critical AND illness) OR (care AND intensive AND unit OR icu)))

Full assessment of risk of bias

Table S1. Risk of bias

Authors	1	2	3	4	5	6	7	8	9	10	11	12	13	14
Fujisawa et al. (2022)	Yes	Yes	Yes	Yes	NA	No	NA	Yes	No	NA	Yes	No	NA	Yes
Haglin et al. (2010)	Yes	Yes	Yes	Yes	NA	NA	NA	Yes	No	NR	Yes	No	NA	No
Heybeli et al. (2022)	No	No	NR	CD	CD	NA	NA	Yes	No	NR	No	No	NA	No
Jang et al. (2022)	Yes	Yes	Yes	Yes	NA	Yes	Yes	Yes	No	No	Yes	No	NA	Yes
Morimoto et al. (2022)	Yes	Yes	CD	Yes	CD	Yes	CD	Yes	No	NR	Yes	No	NA	Yes
Park et al. (2019)	Yes	Yes	NA	Yes	NA	Yes	Yes	Yes	No	NA	Yes	No	No	Yes
Pourhassan et al. (2018)	Yes	Yes	CD	Yes	CD	NA	NA	Yes	No	NA	Yes	No	NA	No
Sankaran et al. (1997)	No	Yes	Yes	No	CD	Yes	Yes	Yes	No	NA	Yes	No	NA	Yes

1. Was the research question or objective in this paper clearly stated? 2. Was the study population clearly specified and defined? 3. Was the participation rate of eligible persons at least 50%? 4. Were all the subjects selected or recruited from the same or similar populations (including the same time period)? Were inclusion and exclusion criteria for being in the study prespecified and applied uniformly to all participants? 5. Was a sample size justification, power description, or variance and effect estimates provided? 6. For the analyses in this paper, were the exposure(s) of interest measured prior to the outcome(s) being measured? 7. Was the timeframe sufficient so that one could reasonably expect to see an association between exposure and outcome if it existed? 8. For exposures that can vary in amount or level, did the study examine different levels of the exposure as related to the outcome (e.g., categories of exposure, or exposure measured as continuous variable)? 9. Were the exposure measures (independent variables) clearly defined, valid, reliable, and implemented consistently across all study participants? 10. Was the exposure(s) assessed more than once over time? 11. Were the outcome measures (dependent variables) clearly defined, valid, reliable, and implemented consistently across all study participants? 12. Were the outcome assessors blinded to the exposure status of participants? 13. Was loss to follow-up after baseline 20% or less? 14. Were key potential confounding variables measured and adjusted statistically for their impact on the relationship between exposure(s) and outcome(s)?
